# Effect of remimazolam on electroencephalogram burst suppression in elderly patients undergoing cardiac surgery: Protocol for a randomized controlled noninferiority trial

**DOI:** 10.1016/j.heliyon.2023.e23879

**Published:** 2023-12-18

**Authors:** Zheng-min Ma, Jing-hui Hu, Yao-yu Ying, Xian Chen, Jing-ya Xu, Wen-wen Huo, Hong Liu, Fu-hai Ji, Ke Peng

**Affiliations:** a Department of Anesthesiology, First Affiliated Hospital of Soochow University, Suzhou, Jiangsu, China; b Institute of Anesthesiology, Soochow University, Suzhou, Jiangsu, China; c Department of Medical Affairs, The Second Affiliated Hospital of Soochow University, Suzhou, China; d Department of Epidemiology and Biostatistics, Soochow University Medical College, Suzhou, China; e Department of Anesthesiology and Pain Medicine, University of California Davis Health, Sacramento, CA, USA

**Keywords:** Remimazolam, Electroencephalogram burst suppression, Postoperative delirium, Cardiac surgery, Elderly patients

## Abstract

**Background:**

Postoperative delirium (POD) is a common complication following cardiac surgery and increases postoperative morbidity and mortality. Intraoperative electroencephalogram (EEG) burst suppression suggests excessively deep anesthesia and predicts POD. Use of remimazolam provides a stable hemodynamic status and an appropriate depth of anesthesia. We aim to assess remimazolam administered for anesthesia and sedation in elderly patients having cardiac surgery.

**Methods:**

This is a randomized controlled clinical trial with noninferiority design. A total of 260 elderly patients aged equal to or greater than 60 years undergoing cardiac surgery will be randomly allocated to receive remimazolam or propofol (1:1) for general anesthesia and postoperative sedation until extubation. The primary outcome is the cumulative time with EEG burst suppression which is obtained from the SedLine system. The noninferiority margin is 2.0 min. The secondary outcomes include the POD occurrence within the first 5 days postoperatively and the duration of perioperative hypotension.

**Discussion:**

This noninferiority trial is the first to evaluate the effect of perioperative remimazolam administration on EEG burst suppression, POD occurrence, and duration of hypotension in elderly patients who undergo cardiac surgery.

**Trial registration:**

Chinese Clinical Trial Registry (ChiCTR2200056353).

## 1 Introduction

Postoperative delirium (POD) is a common complication following surgery with the characteristics of acute dysfunction in attention and awareness [1]. Patients with POD have an increased risk for prolonged hospitalization, dementia, and mortality [2]. The predisposing and precipitating factors of POD include advanced age, electroencephalogram (EEG) burst suppression, cardiac surgery, and admission to intensive care unit (ICU) [[3], [4], [5]]. Patients having cardiac surgery show a high incidence of POD ranging from 10 % to 50 %, and its prevention and treatment remain challenging [6,7].

The indices calculated by EEG monitors such as bispectral index have become widely used to assess the depth of anesthesia and sedation; however, these indices characterize only partial raw EEG features [[8], [9], [10]]. In the recent years, multimodal brain monitoring has been applied to provide individualized anesthesia, illustrating real-time EEG waveforms and derived parameters (density spectral array, spectral edge frequency [SEF], patient state index [PSI], electromyography, and suppression ratio) [11]. Recent studies showed that the utilization of EEG monitoring detected burst suppression and may improve perioperative neurocognitive function [12,13].

Remimazolam is an ultra-short γ-aminobutyric acid type receptor agonist that provides sedation with quick onset and rapid metabolism [14]. The use of remimazolam led to safe and effective sedation for endoscopic procedures [[15], [16], [17], [18]]. Regarding its efficacy for general anesthesia, remimazolam was not inferior to propofol [19]. For patients undergoing cardiovascular surgery, remimazolam reduced cardiac depression and adverse events compared with propofol or etomidate [[20], [21], [22], [23]].

We design this randomized controlled noninferiority trial on remimazolam anesthesia in elderly patients undergoing cardiac surgery. We hypothesize that anesthesia and sedation using remimazolam would be noninferior to propofol in terms of EEG burst suppression. In addition, we will assess the POD occurrence and hypotension duration in these patients.

## 2 Methods

### 2.1 Study design

**Fig. 1:**
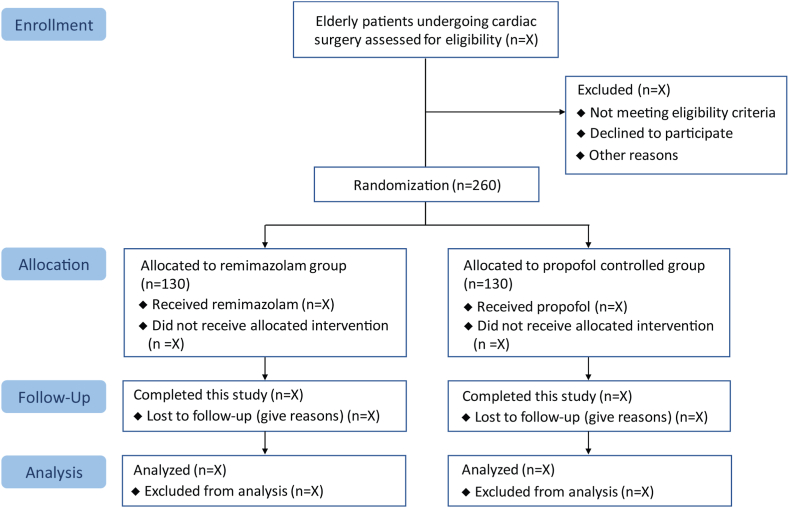
Study flow diagram.

This randomized controlled noninferiority trial is conducted at the First Affiliated Hospital of Soochow University. A total of 260 elderly patients who are scheduled for cardiac surgery will be recruited. After the Medical Ethics Committee approved this trial (No. 2021–303), we completed the registration at the Chinese Clinical Trial Registry (identiﬁer: ChiCTR2200056353). We plan to recruit all patients by the end of December 2024. Written informed consent will be obtained. We report this trial protocol in accordance to the Standard Protocol Items: Recommendations for Interventional Trials (SPIRIT) guideline [24]. Table 1 presents the scheduled process of enrollment, interventions, and measurements. Fig. 1 shows the flowchart of patients in this trial.

**Table 1:** Schedule of patient enrollment, study interventions, and measurements complying with the SPIRIT statement.

Timepoint	Study Period					
	Enrollment	Allocation	Post-allocation			Close-out
	*Pre-anesthesia visit*	Before surgery	During surgery	postoperative day 1 to 5	Hospital discharge	Postoperative day 30
**Enrollment**						
Inclusion criteria	**×**					
Exclusion criteria	**×**					
Written informed consent	**×**					
Randomization		**×**				
Allocation		**×**				
**Interventions**						
Remimazolam			**×**	**×** (until extubation)		
Propofol			×	× (until extubation)		
**Measurements**						
Electroencephalogram data			**×**			
Intraoperative awareness			**×**			
Duration of hypotension			**×**	**×**		
Use of vasopressors/inotropes			**×**	**×** **×**		
Postoperative delirium				**×** **×**		
PONV				**×**		
Postoperative complications					**×**	
Reoperation				**×**	**×**	
Mechanical ventilation					**×**	
Length of ICU stays					**×**	
Length of hospital stays					**×**	
30-day readmission						**×**
30-day mortality						**×**

According to SPIRIT statement of defining standard protocol items for clinical trials.

PONV, postoperative nausea and vomiting.

### 2.2 Eligibility criteria

The inclusion criteria are (1) aged equal to or greater than 60 years, (2) American Society of Anesthesiologists (ASA) classification I to IV, (3) planned to have elective cardiac surgery, and (4) with planned ICU admission. The cardiac procedures include coronary artery bypass grafting and/or valve surgery under cardiopulmonary bypass (CPB).

Exclusion criteria are (1) allergy to study medications, (2) surgical procedures involving aortic arch, (3) use of deep hypothermic circulatory arrest or low flow; (4) preoperative left ventricular ejection fraction <30 %, (5) severe hepatic dysfunction (Child-Pugh class C) or renal replacement therapy, (6) severe chronic respiratory disease, (7) history of mental diseases or taking antipsychotic medications, (8) Mini-Mental State Examination (MMSE) score ≤23, or (9) declined to provide informed consent.

### 2.3 Randomization and blinding

A research assistant not involved in the other parts of the trial generates the random sequence with the use of online randomization (https://www.91trial.com; 1:1, permuted blocks of size 2 or 4). Allocation concealment will be protected by password. According to the randomization results, another research stuff assigns patients into one of the two study groups (the remimazolam group and the propofol group). As remimazolam and propofol have different appearances, the perioperative care team will not be blinded to the study groups; however, they are unaware of the study hypothesis. All patients, data collectors, outcome assessors, and a statistician will be masked to the assignment.

### 2.4 Anesthesia

Patients will receive an ASA routine monitoring. Hemodynamic status will be monitored using the FloTrac/Vigileo (Edwards Lifesciences, CA) via radial artery cannulation. Multimodal brain monitoring with the SedLine® (Masimo, CA) system will be applied to monitor intraoperative EEG continuously. Attending anesthesiologists will receive full education of the characteristic EEG patterns in the SedLine system during propofol and remimazolam anesthesia.

To induce general anesthesia, patients will be administered with intravenous sufentanil 0.5 μg/kg and one of the study medications (remimazolam or propofol, with details described below). Rocuronium 0.8 mg/kg will be given for endotracheal intubation. To maintain anesthesia, patients will receive an infusion of remimazolam or propofol, titrated to an appropriate depth of anesthesia. Intermittent sufentanil 0.1 μg/kg will be given to achieve intraoperative analgesia, and rocuronium 0.1 mg/kg will be administered for neuromuscular block. Patients will receive intraoperative mechanical ventilation (tidal volume 6–8 ml/kg, 12–18 breaths per minute, 50–80 % of oxygen in air) and a standardized normothermic CPB (a continuous nonpulsatile perfusion of 2–2.4 l/min/m^2^). Patients will receive erythrocyte transfusion when hematocrit is ≤ 21 % on CPB and ≤24 % off CPB. Vasoactive and inotropic drugs (phenylephrine, epinephrine, norepinephrine, nitroglycerine, or dobutamine) will be used at the discretion of attending anesthesiologists. To provide prophylaxis of nausea and vomiting, intravenous dexamethasone 5 mg and palonosetron 0.075 mg will be given. Upon the completion of surgery, patients will be admitted to a designated cardiac ICU and receive sedation with a continuous infusion of the study medications until extubation.

### 2.5 Study interventions

**Fig. 2:**
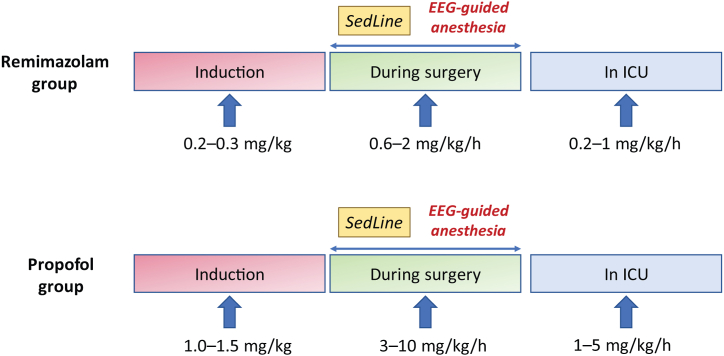
Details of study interventions.

Fig. 2 illustrates the details of study interventions. Patients in the remimazolam group will receive intravenous remimazolam 0.2–0.3 mg/kg to induce anesthesia, an infusion at 0.6–2 mg/kg/h to maintain anesthesia, and 0.2–1 mg/kg/h for sedation until extubation in the ICU. Patients in the propofol group will receive intravenous propofol 1.0–1.5 mg/kg for anesthesia induction, an infusion at 3–10 mg/kg/h during surgery, and sedation with 1–5 mg/kg/h until extubation. The infusion rate of study medications in both groups will be adjusted according to the observation of EEG information. The primary target is to minimize EEG burst suppression episodes by decreasing propofol or remimazolam administration. The secondary targets are to maintain left and right SEF values of 8–15 and PSI values of 25–50 [25]. Other intravenous hypnotic agents (such as midazolam, etomidate, dexmedetomidine, or ketamine) or inhalational agents (such as sevoflurane, isoflurane, or nitrous oxide) will not be used.

### 2.6 Outcomes

For this noninferiority trial, the primary outcome is the cumulative time with EEG burst suppression, which is obtained from the SedLine system [26]. In addition, we will present the adjusted time with burst suppression (time spent in EEG burst suppression divided by surgical time).

The secondary outcomes include (1) the POD occurrence within the first 5 days postoperatively and (2) duration of perioperative hypotension (a decrease in mean arterial pressure >30 % of baseline which requires interventions). POD will be assessed using the Confusion Assessment Method (CAM) or CAM-ICU at 8:00 and 20:00 in the first five postoperative days. The assessment is based on 4 features: acute onset of mental state fluctuation, inattention, disorganized thinking, and consciousness alterations [27]. Patients presenting both features 1 and 2 with either feature 3 or 4 will be diagnosed as delirious [28,29].

Exploratory outcomes include time with PSI <25, time with SEF <8, intraoperative awareness, perioperative use of vasopressors and inotropes, postoperative complications (including myocardial infarction, heart block, new-onset of atrial fibrillation, respiratory failure, pneumonia, stroke, coma, acute kidney injury, renal replacement therapy, and surgical site infection), postoperative nausea and vomiting, reoperation, mechanical ventilation, duration of stay in ICU, length of postoperative hospital stay, 30-d readmission, and 30-d mortality.

### 2.7 Data management

We will collect the demographic data (age, gender, body mass index, smoking status, and education years), baseline data (ASA physical status, comorbidities, preoperative medications, and MMSE score), and perioperative data (surgical type, dose of anesthetics and analgesics, anesthesia time, surgical time, duration of CPB, transfusions). EEG information (left and right SEF, PSI, and burst suppression) will be recorded in SedLine and then data will be analyzed offline. Trained investigators will assess the occurrence of POD using the CAM-ICU or CAM postoperatively. All data will be de-identified and then registered on the web-based database (https://www.91trial.com). A data monitoring committee independent of the study team will conduct an ongoing review of study implementation.

### 2.8 Sample size

According to our institutional pilot data, the mean ± standard deviation (SD) time with EEG burst suppression was 10 ± 5.5 min in elderly patients who received cardiac surgery and propofol anesthesia. We set a noninferiority margin of 2 min based on clinical expert advice. To test the noninferiority of remimazolam with α = 0.025 and power = 80 %, 240 patients are required (n = 120 in each group). We consider a loss to follow-up rate of 8 %, and the target recruitment is set at 260 elderly patients (n = 130 in each group). We calculated the sample size using PASS (version 11.0.7, NCSs, LLC).

### 2.9 Statistical analysis

Continuous variables will be shown as mean ± SD or medians with interquartile range (IQR), and categorical variables as numbers (%). The two groups will be compared using the student's *t*-test, Mann-Whitney *U* test, χ^2^ test, or Fisher's exact test. The interventional treatment effects of remimazolam vs. propofol will be analyzed using the appropriate effect size (i.e., odds ratio, mean difference, or median difference) with the 95 % confidence intervals (CI). With a noninferiority margin of 2 min, noninferiority of remimazolam can be concluded if the upper boundary of 95%CI for the difference in EEG burst suppression time <2.0 min. For the two secondary outcomes, multiple comparison correction with the Bonferroni approach will be performed, and a significance level of *P* < 0.025 will be applied. For the exploratory outcomes, no multiple comparison corrections will be done. Subgroup analyses will be performed according to age, ASA classification, anesthesia time, and CPB time.

The primary analysis will be conducted in the modified intention-to-treat patient population (i.e., all patients who undergo randomization, receive planned cardiac surgery, and have available outcome data) and in the per-protocol population (i.e., all patients receiving the allocated treatment according to the protocol). We do not have plans for interim analysis or missing data imputation. The SPSS (version 26.0, SPSS Inc) will be used for statistical analyses.

## 3 Discussion

We will recruit a total of 260 elderly patients undergoing cardiac surgery in this randomized controlled noninferiority trial to assess remimazolam vs. propofol for anesthesia and postoperative sedation. The primary hypothesis is that remimazolam would be noninferior to propofol in terms of the cumulative time of EEG burst suppression. We also plan to investigate the impact of remimazolam on the POD occurrence and duration of hypotension in these patients.

Recent studies have shown the relationship between postoperative cognitive function and EEG burst suppression. The characteristic pattern of EEG burst suppression is isoelectric periods with high-frequency and large-amplitude waves, suggesting an excessive depth of anesthesia or low cerebral perfusion [30]. An observational study comprising 619 surgical patients with planned admission to ICU showed that intraoperative EEG suppression could predict POD [31]. This association was also observed in patients undergoing cardiac surgery [32,33]. However, a clinical trial showed that anesthesia administration by EEG guidance reduced the cumulative time with EEG suppression but did not decrease POD in elderly patients who had major surgery [34]. Therefore, more efforts are still required to determine whether the avoidance of EEG burst suppression reduces POD.

A previous study showed that the time with burst suppression was 10.9 ± 2.6 min in patients who received neurosurgical procedures and propofol anesthesia [35]. A recent study showed that for patients receiving sevoflurane anesthesia and major surgery, the median (IQR) time with EEG suppression was 7 (1–23) min in the guided group and 13 (2–58) min in the control group, accounting for 2.7 % and 4.9 % of total anesthesia time respectively. However, there is no report on the frequency or cumulative time of EEG burst suppression during remimazolam anesthesia. In our pilot observation, time with EEG burst suppression was 10 ± 5.5 min in cardiac surgical patients who received propofol anesthesia. In this randomized controlled noninferiority trial, we set a margin of 2 min to test the noninferiority of remimazolam.

Remimazolam is a new type of benzodiazepine administered for sedation and anesthesia. Recent studies suggested that the pharmacokinetics of remimazolam was not influenced by the baseline factors such as age, sex, and weight [36], without accumulation or prolonged efficacy even after a long-term or high-dose infusion [37]. Duan et al. enrolled 60 elderly patients who had hip replacement to show that remimazolam anesthesia, when compared with propofol anesthesia, could reduce the incidence of emergence agitation [38]. Another recent study found that remimazolam anesthesia did not increase POD among elderly patients undergoing orthopedic surgical procedures [39]. However, the role of remimazolam in cardiac surgery and its impact on POD are largely unknown.

This study will be the first to assess remimazolam anesthesia in elderly patients who have cardiac surgery. Our primary focus is intraoperative EEG burst suppression, and we also observe POD and hypotension. The limitations of this trial are as follows. First, we select the dosage of remimazolam according to previous studies and our institutional experience, and its optimal dosage in cardiac surgery needs further investigations. Nonetheless, we will utilize the multimodal EEG monitoring to guide an individualized administration of remimazolam or propofol. Second, postoperative care is not controlled, which may confound the study results. Although that may affect the occurrence of POD, our primary outcome (intraoperative EEG burst suppression duration) is an objective parameter obtained by the SedLine monitor.

In conclusion, we conduct this randomized noninferiority trial to explore the effects of remimazolam on EEG burst suppression, prevalence of POD, and duration of hypotension for older patients undergoing cardiac surgery. The findings from this trial will provide clinical evidence of remimazolam administration and patient outcomes in cardiac surgery.

## 4 Ethics statement

Ethical approval was obtained from the Ethics Committee of the First Affiliated Hospital of Soochow University (No. 2021–303) on Jan 6, 2022. A follow-up review was carried out on Jun 1, 2023 (No. 2021-303-1). All patients will provide written informed consent. Personal information will be kept confidential.

## Funding

This work will be supported by 10.13039/501100012219Jiangsu Medical Association Anesthesia Research Project (SYH-32021-0036 (2021031)), Suzhou Medical Health Science and Technology Innovation Project (SKY2022136), 10.13039/501100004608Natural Science Foundation of Jiangsu Province (BK20200195), 10.13039/501100005066Key Medical Research Projects in Jiangsu Province (ZD2022021), and Suzhou Clinical Medical Center for Anesthesiology (Szlcyxzxj202102).

## Data availability statement

No data is used. This manuscript is a study protocol. The patient recruitment is ongoing. The data will be available from the corresponding author upon reasonable request.

## CRediT authorship contribution statement

**Zheng-min Ma:** Writing – original draft, Resources, Methodology, Investigation, Formal analysis, Conceptualization. **Jing-hui Hu:** Writing – original draft, Visualization, Software, Resources, Methodology, Investigation, Formal analysis, Conceptualization. **Yao-yu Ying:** Writing – original draft, Visualization, Software, Resources, Methodology, Formal analysis, Data curation. **Xian Chen:** Writing – original draft, Validation, Investigation, Data curation. **Jing-ya Xu:** Writing – original draft, Visualization, Software, Investigation, Data curation. **Wen-wen Huo:** Writing – review & editing, Visualization, Validation, Methodology, Funding acquisition, Formal analysis. **Hong Liu:** Writing – review & editing, Validation, Supervision, Methodology, Conceptualization. **Fu-hai Ji:** Writing – review & editing, Validation, Supervision, Funding acquisition, Conceptualization. **Ke Peng:** Writing – review & editing, Validation, Supervision, Software, Resources, Methodology, Funding acquisition, Conceptualization.

## Declaration of competing interest

The authors declare that they have no known competing financial interests or personal relationships that could have appeared to influence the work reported in this paper.

## References

[1] Wilson J.E., Mart M.F., Cunningham C. (2020). Delirium. Nat Rev Dis Primers.

[2] Witlox J., Eurelings L.S., de Jonghe J.F., Kalisvaart K.J., Eikelenboom P., van Gool W.A. (2010). Delirium in elderly patients and the risk of postdischarge mortality, institutionalization, and dementia: a meta-analysis. JAMA.

[3] Ormseth C.H., LaHue S.C., Oldham M.A., Josephson S.A., Whitaker E., Douglas V.C. (Jan 3 2023). Predisposing and precipitating factors associated with delirium: a systematic review. JAMA Netw. Open.

[4] Bramley P., McArthur K., Blayney A., McCullagh I. (Sep 2021). Risk factors for postoperative delirium: an umbrella review of systematic reviews. Int. J. Surg..

[5] Ho M.H., Nealon J., Igwe E. (Oct 2021). Postoperative delirium in older patients: a systematic review of assessment and incidence of postoperative delirium. Worldviews Evidence-Based Nurs..

[6] Cai S., Li J., Gao J., Pan W., Zhang Y. (Dec 2022). Prediction models for postoperative delirium after cardiac surgery: systematic review and critical appraisal. Int. J. Nurs. Stud..

[7] de la Varga-Martinez O., Gomez-Pesquera E., Munoz-Moreno M.F. (May 2021). Development and validation of a delirium risk prediction preoperative model for cardiac surgery patients (DELIPRECAS): an observational multicentre study. J. Clin. Anesth..

[8] Radtke F.M., Franck M., Lendner J., Kruger S., Wernecke K.D., Spies C.D. (Jun 2013). Monitoring depth of anaesthesia in a randomized trial decreases the rate of postoperative delirium but not postoperative cognitive dysfunction. Br. J. Anaesth..

[9] Sieber F.E., Zakriya K.J., Gottschalk A. (Jan 2010). Sedation depth during spinal anesthesia and the development of postoperative delirium in elderly patients undergoing hip fracture repair. Mayo Clin. Proc..

[10] Chan M.T., Cheng B.C., Lee T.M., Gin T., Group C.T. (Jan 2013). BIS-guided anesthesia decreases postoperative delirium and cognitive decline. J. Neurosurg. Anesthesiol..

[11] Hight D., Kreuzer M., Ugen G. (May 2023). Five commercial 'depth of anaesthesia' monitors provide discordant clinical recommendations in response to identical emergence-like EEG signals. Br. J. Anaesth..

[12] Yang S., Xiao W., Wu H. (2021). Management based on multimodal brain monitoring may improve functional connectivity and post-operative neurocognition in elderly patients undergoing spinal surgery. Front. Aging Neurosci..

[13] Xu N., Li L.X., Wang T.L. (2021). Processed multiparameter electroencephalogram-guided general anesthesia management can reduce postoperative delirium following carotid endarterectomy: a randomized clinical trial. Front. Neurol..

[14] Lee A., Shirley M. (Jul 2021). Remimazolam: a review in procedural sedation. Drugs.

[15] Chen S.H., Yuan T.M., Zhang J. (Feb 2021). Remimazolam tosilate in upper gastrointestinal endoscopy: a multicenter, randomized, non-inferiority, phase III trial. J. Gastroenterol. Hepatol..

[16] Pastis N.J., Yarmus L.B., Schippers F. (Jan 2019). Safety and efficacy of remimazolam compared with placebo and midazolam for moderate sedation during bronchoscopy. Chest.

[17] Wang X., Hu X., Bai N. (2022). Safety and efficacy of remimazolam besylate in patients undergoing colonoscopy: a multicentre, single-blind, randomized, controlled, phase Ⅲ trial. Front. Pharmacol..

[18] Dou D., Feng Y., Jiang L. (Mar 2022). Efficiency and safety of remimazolam and midazolam in digestive endoscopic sedation: systematic review and meta-analysis. Dig. Endosc..

[19] Doi M., Morita K., Takeda J., Sakamoto A., Yamakage M., Suzuki T. (Aug 2020). Efficacy and safety of remimazolam versus propofol for general anesthesia: a multicenter, single-blind, randomized, parallel-group, phase IIb/III trial. J. Anesth..

[20] Gao J., Yang C., Ji Q., Li J. (May 4 2023). Effect of remimazolam versus propofol for the induction of general anesthesia on cerebral blood flow and oxygen saturation in elderly patients undergoing carotid endarterectomy. BMC Anesthesiol..

[21] Liu T., Lai T., Chen J. (Oct 2021). Effect of remimazolam induction on hemodynamics in patients undergoing valve replacement surgery: a randomized, double-blind, controlled trial. Pharmacol Res Perspect.

[22] Kaneko S., Morimoto T., Ichinomiya T., Murata H., Yoshitomi O., Hara T. (Apr 2023). Effect of remimazolam on the incidence of delirium after transcatheter aortic valve implantation under general anesthesia: a retrospective exploratory study. J. Anesth..

[23] Hu B., Zhang M., Wu Z. (2023). Comparison of remimazolam tosilate and etomidate on hemodynamics in cardiac surgery: a randomised controlled trial. Drug Des. Dev. Ther..

[24] Calvert M., Kyte D., Mercieca-Bebber R. (Feb 6 2018). Guidelines for inclusion of patient-reported outcomes in clinical trial protocols: the SPIRIT-PRO extension. JAMA.

[25] Li Y., Bohringer C., Liu H. (Oct 2020). Double standard: why electrocardiogram is standard care while electroencephalogram is not?. Curr. Opin. Anaesthesiol..

[26] Tang C.J., Jin Z., Sands L.P. (Oct 2020). ADAPT-2: a randomized clinical trial to reduce intraoperative EEG suppression in older surgical patients undergoing major noncardiac surgery. Anesth. Analg..

[27] Oh E.S., Fong T.G., Hshieh T.T., Inouye S.K. (Sep 26 2017). Delirium in older persons: advances in diagnosis and treatment. JAMA.

[28] Ely E.W., Inouye S.K., Bernard G.R. (Dec 5 2001). Delirium in mechanically ventilated patients: validity and reliability of the confusion assessment method for the intensive care unit (CAM-ICU). JAMA.

[29] Sauer A.C., Veldhuijzen D.S., Ottens T.H., Slooter A.J.C., Kalkman C.J., van Dijk D. (Aug 1 2017). Association between delirium and cognitive change after cardiac surgery. Br. J. Anaesth..

[30] Hagihira S. (Jul 2015). Changes in the electroencephalogram during anaesthesia and their physiological basis. Br. J. Anaesth..

[31] Fritz B.A., Kalarickal P.L., Maybrier H.R. (Jan 2016). Intraoperative electroencephalogram suppression predicts postoperative delirium. Anesth. Analg..

[32] Soehle M., Dittmann A., Ellerkmann R.K., Baumgarten G., Putensen C., Guenther U. (Apr 28 2015). Intraoperative burst suppression is associated with postoperative delirium following cardiac surgery: a prospective, observational study. BMC Anesthesiol..

[33] Pedemonte J.C., Plummer G.S., Chamadia S. (Aug 2020). Electroencephalogram burst-suppression during cardiopulmonary bypass in elderly patients mediates postoperative delirium. Anesthesiology.

[34] Wildes T.S., Mickle A.M., Ben Abdallah A. (Feb 5 2019). Effect of electroencephalography-guided anesthetic administration on postoperative delirium among older adults undergoing major surgery: the ENGAGES randomized clinical trial. JAMA.

[35] Illievich U., Petricek W., Schramm W., Weindlmayr-Goettel M., Czech T., Spiss C. (1993). Electroencephalographic burst suppression by propofol infusion in humans: hemodynamic consequences. Anesth. Analg..

[36] Schuttler J., Eisenried A., Lerch M., Fechner J., Jeleazcov C., Ihmsen H. (Apr 2020). Pharmacokinetics and pharmacodynamics of remimazolam (CNS 7056) after continuous infusion in healthy male volunteers: Part I. Pharmacokinetics and clinical pharmacodynamics. Anesthesiology.

[37] Wang M., Zhao X., Yin P., Bao X., Tang H., Kang X. (2022). Profile of remimazolam in Anesthesiology: a narrative review of clinical research progress. Drug Des. Dev. Ther..

[38] Duan J., Ju X., Wang X., Liu N., Xu S., Wang S. (2023). Effects of remimazolam and propofol on emergence agitation in elderly patients undergoing hip replacement: a clinical, randomized, controlled study. Drug Des. Dev. Ther..

[39] Yang J.J., Lei L., Qiu D. (2023). Effect of remimazolam on postoperative delirium in older adult patients undergoing orthopedic surgery: a prospective randomized controlled clinical trial. Drug Des. Dev. Ther..

